# Supplementary material for: BRG1 attenuates colonic inflammation and tumorigenesis through autophagy-dependent oxidative stress sequestration
Source: Nat Commun. 2019 Oct 10;10:4614. doi: 10.1038/s41467-019-12573-z (PMC6787222; doi:10.1038/s41467-019-12573-z)
Supplement: Supplementary file 1 — Supplementary Information [file 41467_2019_12573_MOESM1_ESM.pdf]

SUPPLEMENTARY INFORMATION

BRG1 Attenuates Colonic Inflammation and Tumorigenesis through Autophagy-dependent Oxidative Stress Sequestration

by Min Liu et.al

Supplementary Figure. 1 Deletion of Brg1 in adult colonic epithelium.

Supplementary Figure. 2 Immunostaining experiments of colons and intestines derived from 1% DSS-treated $Brg1^{F/F}$ and $Brg1^{IEC\text{-}AKO}$ mice.

Supplementary Figure. 3 BRG1 overexpression does not affect the self-renew and differentiation of IECs under steady state.

Supplementary Figure. 4 Increase ROS leads to colitis in $Brg1^{IEC\text{-}AKO}$ mice.

Supplementary Figure. 5 BRG1 modulates autophagy in colonic epithelial cells.

Supplementary Figure. 6 Analyses in the $R26^{Brg1+}$ and $Brg1^{IEC\text{-}OE/+}$ mice with or without Atg5 deletion in IECs.

Supplementary Figure. 7 Analyses in the $Brg1^{F/F}$ and $Brg1^{IEC\text{-}AKO}$ mice with or without antibiotics treatment.

Supplementary Figure. 8 Adult Brg1 loss does not affect Notch pathway in the colons.

Supplementary Table 1 Primers.

Supplementary Figure 1

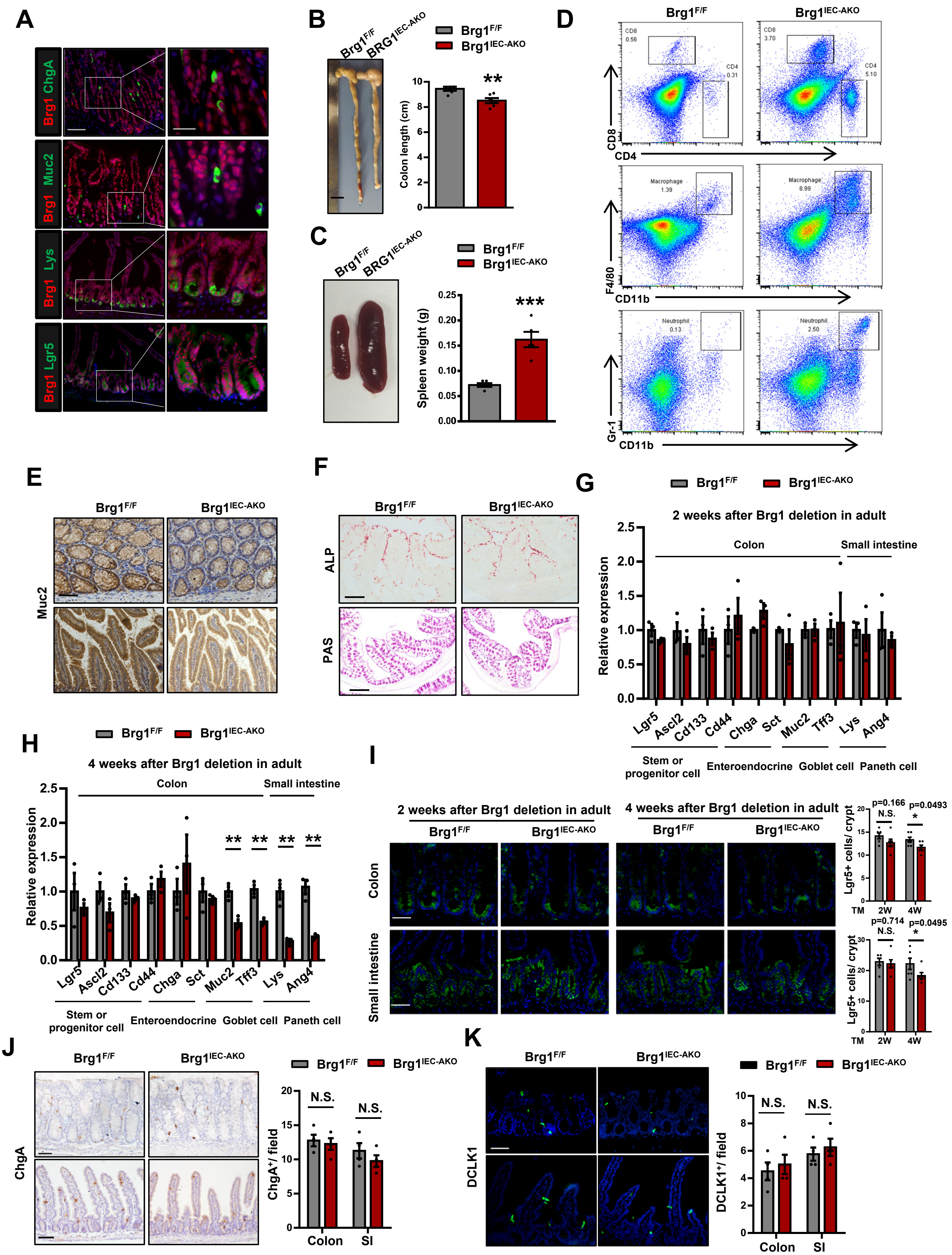

Supplementary Figure. 1 Deletion of Brg1 in adult colonic epithelium.

(a) Co-immunofluorescence staining of BRG1 with Lgr5, ChgA, Muc2, and Lys in intestine and colon of wild-type mice.

(b) Colon length of 4-month-old Brg1$^{F/F}$ and Brg1$^{IEC\text{-}AKO}$ mice (two months after Brg1 depletion).

(c) Spleen weight of 4-month-old Brg1$^{F/F}$ and Brg1$^{IEC\text{-}AKO}$ mice (two months after Brg1 depletion).

(d) Representative flow cytometry plots of CD4+ T cells, CD11b+; F4/80+ macrophages, and CD11b+; Gr-1+ neutrophils.

(e) Immunostaining of Muc2 in colons and intestines of 3-month-old Brg1$^{F/F}$ and Brg1$^{IEC\text{-}AKO}$ mice (4 weeks after Brg1 depletion).

(f) ALP (Enterocyte) and PAS (Goblet cells) staining of colon sections after 2 weeks of Brg1 deletion.

(g, h) RT-qPCR analysis of gene expressions in the intestines of Brg1$^{F/F}$ and BRG1$^{IEC\text{-}AKO}$ mice at the indicated time points.

(i) Immunofluorescent staining of Lgr5 in colons and intestines of Brg1$^{F/F}$ and BRG1$^{IEC\text{-}AKO}$ mice.

(j, k) Immunostaining of ChgA **(j)** and DCLK1**(k)** in colons and intestines of 3-month-old Brg1$^{F/F}$ and Brg1$^{IEC\text{-}AKO}$ mice and quantitation results are shown at the bottom. Data represent mean ± S.E.M.; * $p < 0.05$; ** $p < 0.01$; *** $p < 0.001$. Scale Bar: 50 μm **left a, e, i, upper j, k**, 20 μm **right a,** 1 cm **b**, 100 μm **f, bottom j**. SI: Small Intestine.

Supplementary Figure 2

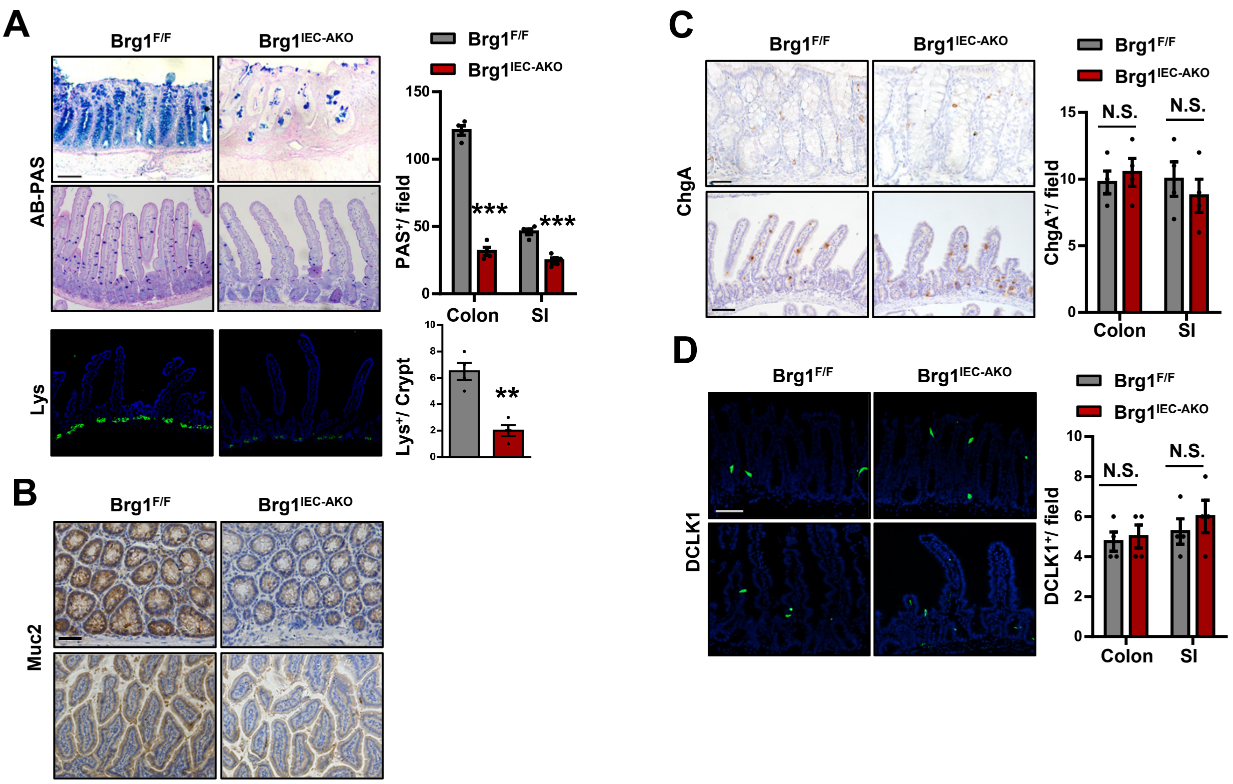

Supplementary Figure. 2 Immunostaining experiments of colons and intestines derived from 1% DSS-treated Brg1$^{F/F}$ and Brg1$^{IEC\text{-}AKO}$ mice.

(a-d) AB-PAS staining and immunostaining of Lys **(a)**, Muc2**(b)**, ChgA **(c)** and DCLK1**(d)** in colons and intestines of 1% DSS-treated Brg1$^{F/F}$ and Brg1$^{IEC\text{-}AKO}$ mice and quantitation results are shown in the right. ** $p < 0.01$ and *** $p < 0.0001$. Scale Bars: 100 μm **a, bottom c**, 50 μm **b, upper c, d**.

Supplementary Figure 3

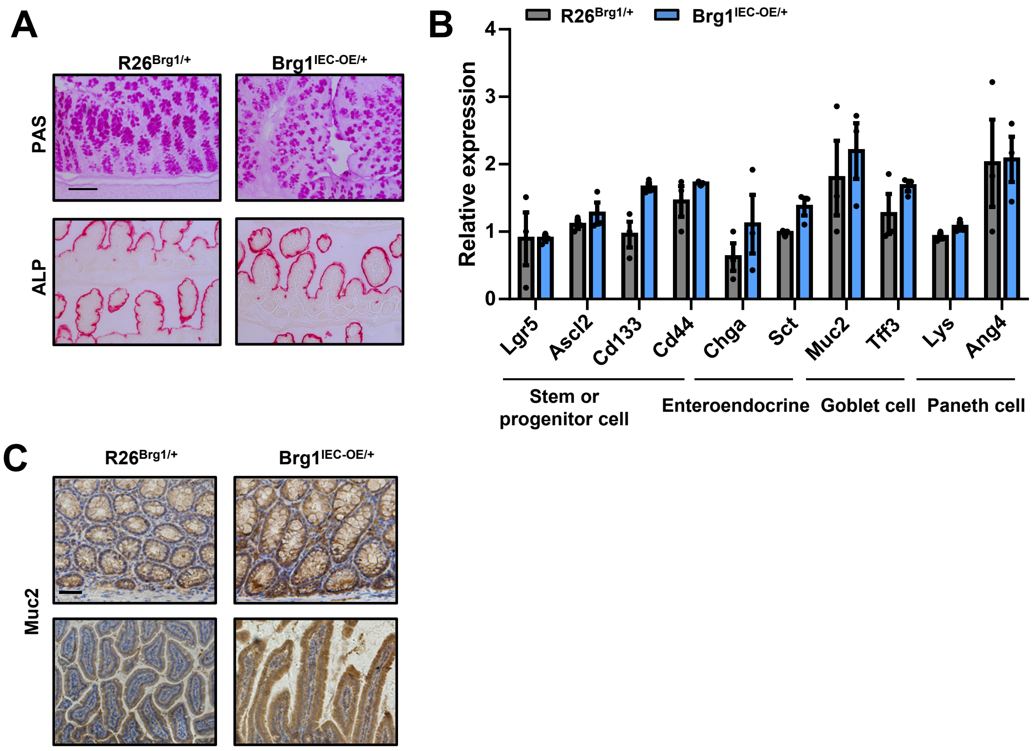

Supplementary Figure. 3 BRG1 overexpression does not affect the self-renew and differentiation of IECs under steady state.

(a) PAS and ALP staining of colon sections as indicated. Scale Bar: 100 μm.

(**b)** RT-qPCR analysis of gene expressions in the colons of R26$^{Brg1/+}$ and Brg1$^{IEC\text{-}OE/+}$ mice as indicated.

(c) Muc2 staining of colon and intestine sections of 3% DSS-treated R26$^{Brg1/+}$ and Brg1$^{IEC\text{-}OE/+}$ mice. Data represent mean ± S.E.M.; * $p < 0.05$; ** $p < 0.01$. Scale Bar: 100 μm **a**. 50 μm **c**.

Supplementary Figure 4

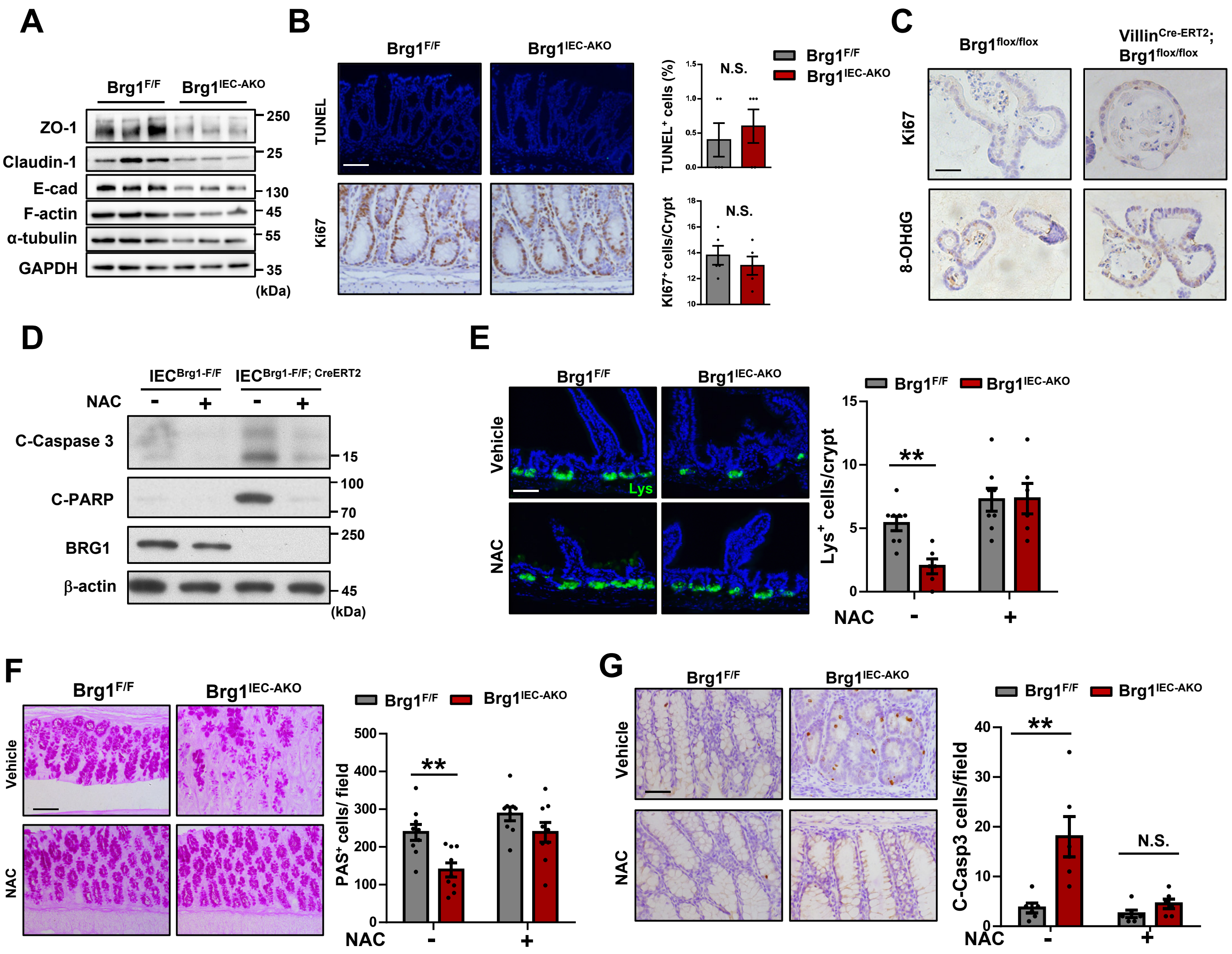

Supplementary Figure. 4 Increase ROS leads to colitis in Brg1$^{IEC\text{-}AKO}$ mice.

(a) Immunoblotting analysis of the indicated protein in IECs isolated from Brg1$^{F/F}$ and Brg1$^{IEC\text{-}AKO}$ mice.

(b) TUNEL and Ki67 staining colon sections derived from 10-week-old Brg1$^{F/F}$ and Brg1$^{IEC\text{-}AKO}$ mice (n = 4).

(c) Ki67 and 8-OHdG staining of the organoid sections as indicated.

(d) IECs were isolated from 1-month-old Brg1$^{flox/flox}$ and Villin$^{Cre\text{-}ERT2}$; Brg1$^{flox/flox}$ mice, and Brg1 deletion is achieved by 4-OHT treatment in cells Western blot analysis of the indicated protein in IEC$^{Brg1\text{-}F/F}$, IEC$^{Brg1F/F\text{-}CreERT2}$ cells with or without NAC(5mM) treatment.

(e-g) Brg1 was knockout at two months of age. Lysosome **(e)**, PAS **(f)** and cleaved caspase-3 staining **(g)** of 4-month-old Brg1$^{F/F}$ and Brg1$^{IEC\text{-}AKO}$ mice with or without NAC treatment (starting at 3 months of age, one month of NAC treatment). Scale Bar: 50 μm **b, e, f, g**. 20 μm **c**. ** $p < 0.01$.

Supplementary Figure 5

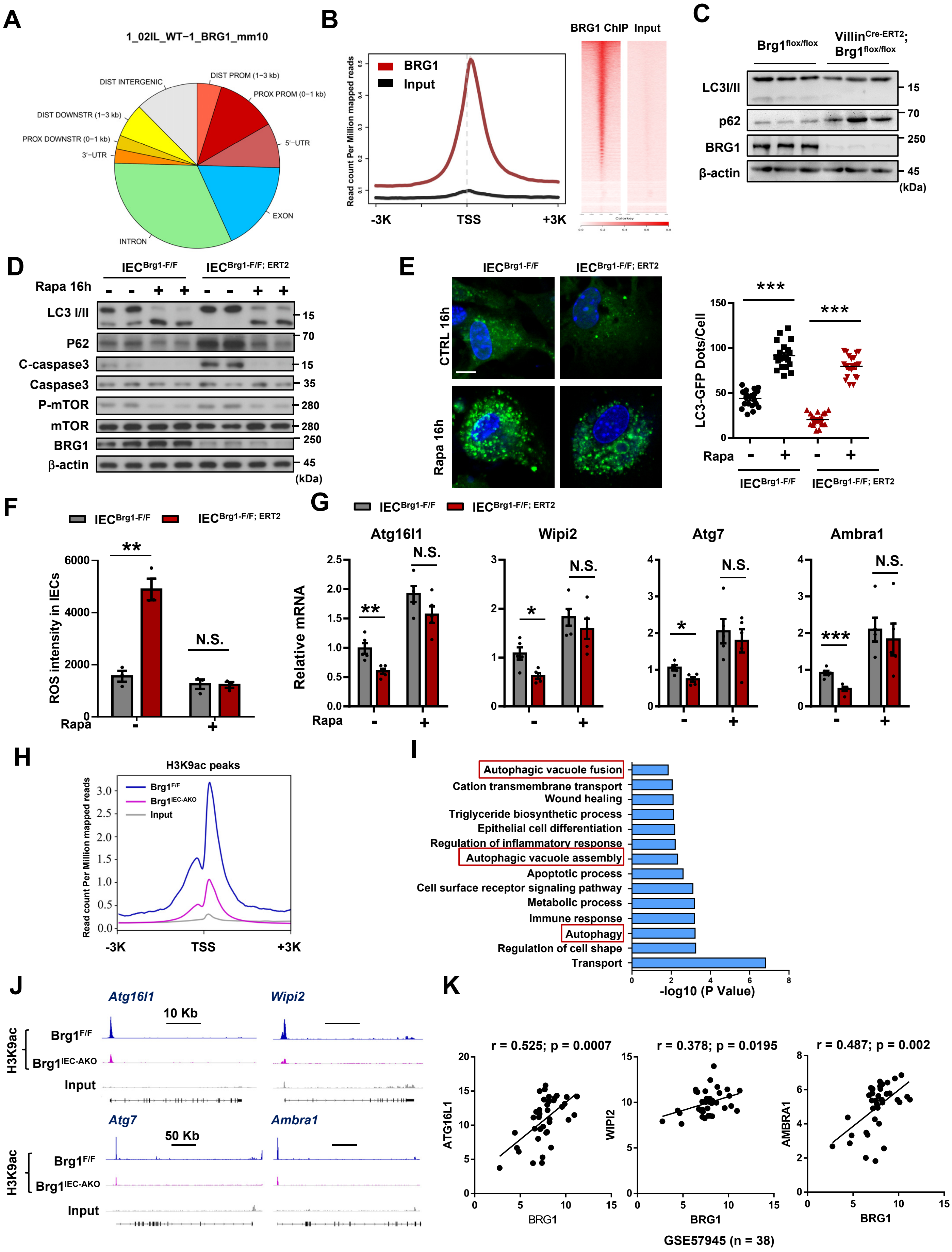

Supplementary Figure. 5 BRG1 modulates autophagy in colonic epithelial cells.

(a) Enrichment distribution for genomic annotations for BRG1 ChIP binding in wild-type IECs.

(**b)** Average BRG1 ChIP signal across 12119 annotated genes in IECs isolated from wild-type mice.

(c) Immunoblotting analysis of the indicated protein in organoids derived from $Brg1^{F/F}$ and $Brg1^{IEC\text{-}AKO}$ mice.

(d-e) IECs were isolated from 2-month-old $Brg1^{flox/flox}$ and $Villin^{Cre\text{-}ERT2}$; $Brg1^{flox/flox}$ mice, and Brg1 deletion is achieved by 4-OHT treatment in cells. After 3 days of culture, western blot analyzes the indicated protein **(d)**, LC3-GFP staining **(e)**.

(f) MFI quantification of ROS in $IEC^{Brg1\text{-}F/F}$ and $IEC^{Brg1\text{-}F/F;\ CreERT2}$ with or without 16 hours of rapamycin treatment.

(g) RT-qPCR analysis of the relative mRNA levels of the indicated genes in $IEC^{Brg1\text{-}F/F}$ and $IEC^{Brg1\text{-}F/F;\ CreERT2}$ with or without 16 hours of rapamycin treatment. (n=5 per genotype).

(h) Average H3K9ac ChIP signal across 12573 annotated genes in IECs isolated from wild-type mice.

(i) The enrichments of GO terms in the overlapping genes of BRG1, H3K9ac binding and displaying expression changes in Brg1-KO IECs.

(j) Snapshot of H3K9ac ChIP-Seq signals at the Atg16l1, Wipi2b, Atg7 and Ambra1 gene loci in IECs isolated from $Brg1^{F/F}$ and $Brg1^{IEC\text{-}AKO}$ mice.

(**k)** The correlation between BRG1 and ATG16L1, WIPI2b or AMBRA1 mRNA in IBD specimens (GSE57945, n = 38) is shown in a regression plot (by Pearson's). * $p < 0.05$; ** $p < 0.01$; *** $p < 0.001$. Scale Bar: 10 μm **e**.

Supplementary Figure 6

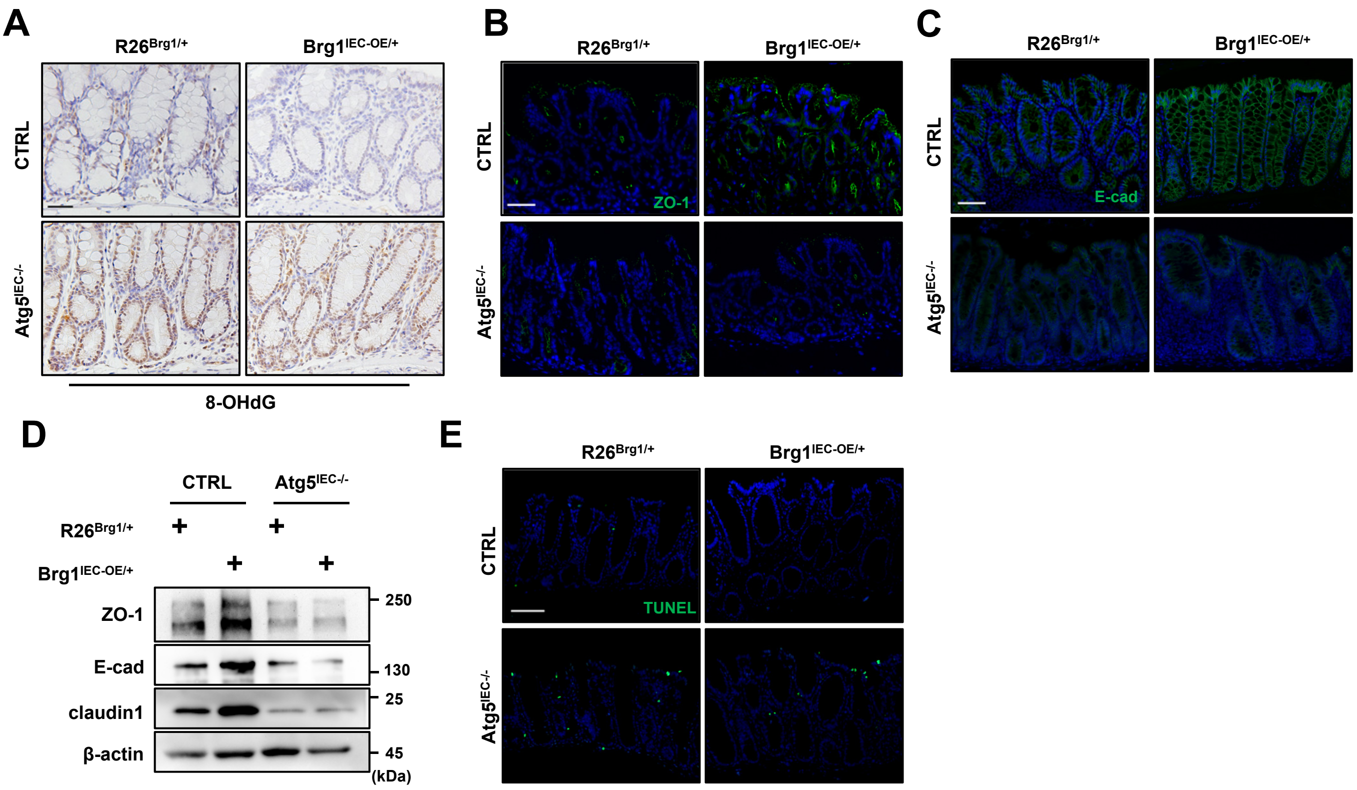

Supplementary Figure. 6 Analyses in the R26^{Brg1+} and Brg1$^{IEC\text{-}OE/+}$ mice with or without Atg5 deletion in IECs.

(a) 8-OHdG staining as indicated.

(b, c) Representative ZO-1 and E-cadherin staining in colon sections as indicated.

(d) Colon lysates were analyzed by western blotting with the indicated antibodies.

(e) TUNEL staining as indicated. Scale Bars: 50 µm a, b, c, e.

Supplementary Figure 7

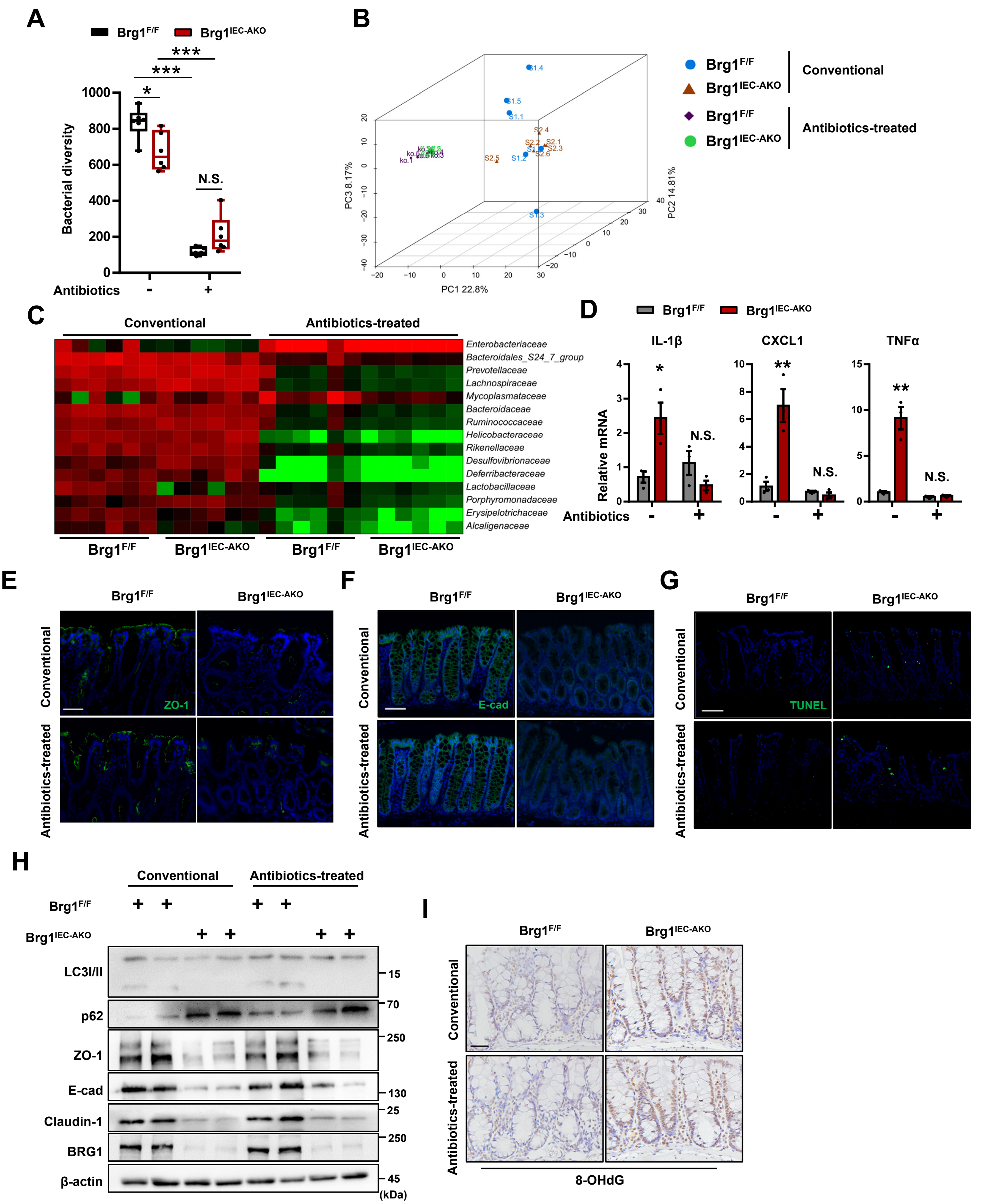

Supplementary Figure. 7 Analyses in the Brg1$^{F/F}$ and Brg1$^{IEC-AKO}$ mice with or without antibiotics treatment.

(a) Chao bacterial diversity in Brg1$^{F/F}$ and Brg1$^{IEC-AKO}$ mice with or without antibiotics treatment.

(b) Analysis of beta diversity between stool samples of Brg1$^{F/F}$ and Brg1$^{IEC-AKO}$ mice.

(c) Heatmap of bacterial family in the intestinal microbiota of Brg1$^{F/F}$ and Brg1$^{IEC-AKO}$ mice.

(d) RT-qPCR analysis of colon homogenates from Brg1$^{F/F}$ and Brg1$^{IEC-AKO}$ mice to assess cytokine and chemokine productions.

(e) and **(f)** Representative ZO-1 and E-cadherin staining in colon sections as indicated.

(g) TUNEL staining as indicated.

(h) Colon lysates were analyzed by western blotting with the indicated antibodies.

(i) 8-OHdG staining as indicated.

* $p < 0.05$; ** $p < 0.001$; *** $p < 0.0001$. Scale Bars: 50 μm e, f, g, i.

Supplementary Figure 8

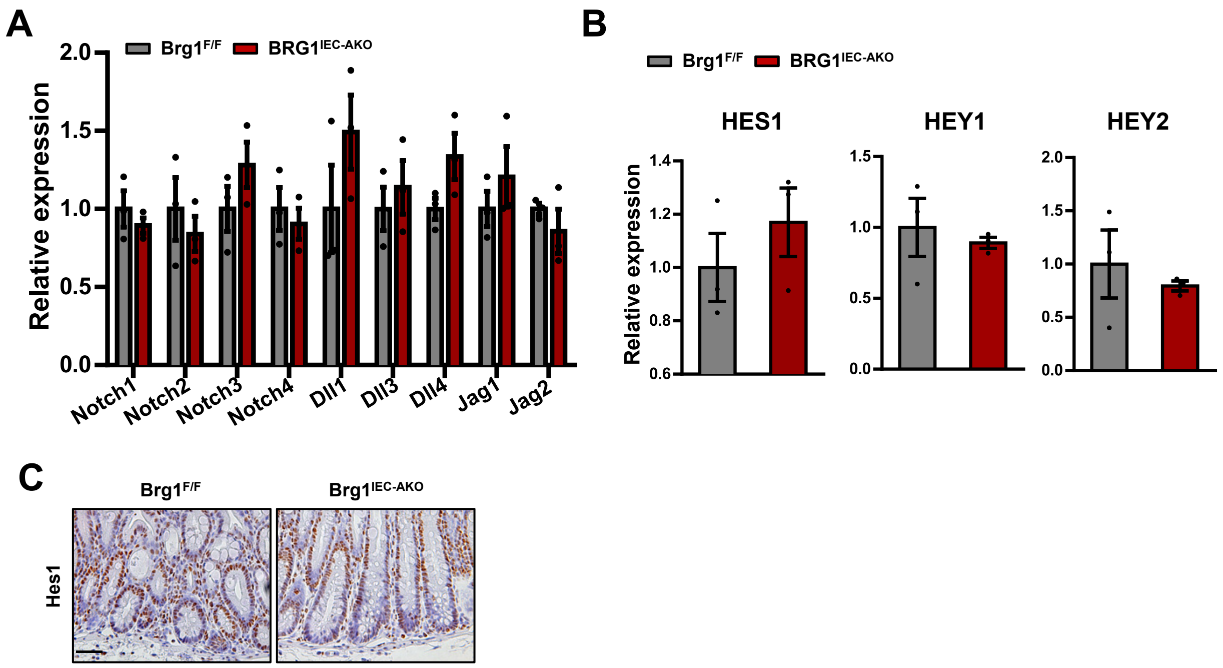

Supplementary Figure. 8 Adult Brg1 loss does not affect Notch pathway in the colons.

(a) RT-qPCR analysis of Notch signaling pathway in the colonic lysates of 3-month-old control and Brg1$^{IEC-AKO}$ mice (one month after Brg1 depletion).

(b) RT-qPCR analysis of Notch targets in the colonic lysates of 3-month-old control and Brg1$^{IEC-AKO}$ mice (one month after Brg1 depletion).

(c) Hes1 staining in colon sections of 3-month-old control and Brg1$^{IEC-AKO}$ mice (one month after Brg1 depletion). Scale Bars: 50 μm c.

Supplementary Table 1

Primers for RT-qPCR

Primer	Sequence(5'-3')	Sequence(3'-5')
**TNFα**	CCCTCACACTCAGATCATCTTCT	GCTACGACGTGGGCTACAG
**Il-6**	TAGTCCTTCCTACCCCAATTTCC	TTGGTCCTTAGCCACTCCTTC
**Cxcl1**	CTGGGATTCACCTCAAGAACATC	CAGGGTCAAGGCAAGCCTC
**Il-1β**	GCAACTGTTCCTGAACTCAACT	ATCTTTTGGGGTCCGTCAACT
**Ccl2**	TTAAAAACCTGGATCGGAACCAA	GCATTAGCTTCAGATTTACGGGT
**Cox2**	GATGCTCTTCCGAGCTGTG	GGATTGGAACAGCAAGGATTT
**Atg16l1**	CAGGCGTTCGAGGAGATCATT	ACTATCATTCCACGCACCATCA
**Wipi2**	AGGATAACACGTCCCTAGCTG	TCTCTCCACAATGCAGACATCT
**Ambra1**	GAGCTGGTGGAGGATAAGACT	GGTCGAGCGTGGACTATCAG
**Atg7**	GTTCGCCCCCTTTAATAGTGC	TGAACTCCAACGTCAAGCGG
**Lgr5**	CCTACTCGAAGACTTACCCAGT	GCATTGGGGTGAATGATAGCA
**Ascl2**	GCCTACTCGTCGGAGGAA	CCAACTGGAAAAGTCAAGCA
**Cd133**	TTGGTGCAAATGTGGAAAAG	ATTGCCATTGTTCCTTGAGC
**Cd44**	AGAAAAATGGCCGCTACAGTATC	TGCATGTTTCAAAACCCTTGC
**ChgA**	CCAAGGTGATGAAGTGCGTC,	GGTGTCGCAGGATAGAGAGGA
**Sct**	AGACACTCAGACGGAATGTTCA	CTGGTCCTCTAAGGGCTTGGA
**Muc2**	TCCACCATGGGGCTGCCACT	GGCCCGAGAGTAGACCTTGG
**Tff3**	CTTTGACTCCAGTATCCCAAATG	TGGCTGTGAGGTCTTTATTCTTC
**Lys**	ATGGAATGGCTGGCTACTATGG	ACCAGTATCGGCTATTGATCTGA
**Ang4**	GGTTGTGATTCCTCCAACTCTG	CTGAAGTTTTCTCCATAAGGGCT
**Notch1**	GATGGCCTCAATGGGTACAAG	TCGTTGTTGTTGATGTCACAGT
**Notch2**	ATGTGGACGAGTGTCTGTTGC	GGAAGCATAGGCACAGTCATC
**Notch3**	TGCCAGAGTTCAGTGGTGG	CACAGGCAAATCGGCCATC
**Notch4**	CTCTTGCCACTCAATTTCCCT	TTGCAGAGTTGGGTATCCCTG
**Dll1**	CAGGACCTTCTTTCGCGTATG	AAGGGGAATCGGATGGGGTT
**Dll3**	CTGGTGTCTTCGAGCTACAAAT	TGCTCCGTATAGACCGGGAC
**Dll4**	TTCCAGGCAACCTTCTCCGA	ACTGCCGCTATTCTTGTCCC
**Jag1**	CCTCGGGTCAGTTTGAGCTG	CCTTGAGGCACACTTTGAAGTA
**Hes1**	CCAGCCAGTGTCAACACGA	AATGCCGGGAGCTATCTTTCT
**Hey1**	GCGCGGACGAGAATGGAAA	TCAGGTGATCCACAGTCATCTG
**Hey2**	AAGCGCCCTTGTGAGGAAAC	GGTAGTTGTCGGTGAATTGGAC
**Axin2**	AACCTATGCCCGTTTCCTCTA	GAGTGTAAAGACTTGGTCCACC
**Apcdd1**	CTTCACGGCGTCCAAGTCAT	GCAAGTTCGGTTCACCAGTC
**Ephb2**	GCGGCTACGACGAGAACAT	GGCTAAGTCAAAATCAGCCTCA
**Nkd1**	AGGAAAGGCATCGAGGAGTG	TCGCTCAGTCTCTCCATTCTC
**Lrig1**	TTGAGGACTTGACGAATCTGC	CTTGTTGTGCTGCAAAAAGAGAG

Primers for ChIP-qPCR

**Atg16l1**	AAGGAGGAAGGAAACAGACAC	AGCCGCCAGCGAACAGAA
**Wipi2**	TGAGACCTGGCCTTTCGC	TGGATTACTACTGCCGTTTGG
**Ambra1**	CAGCAGGAGCTGAGCAATG	AAGAAGACGCCGCAAAA
**Atg7**	GAATGAGCAACCAGAGGC	AGTTGAGCGGCGGTAAGT

Oligos for siRNA

**siAtg16l1-1**	GUCAACUACAAGAAAUGGCTT	GCCAUUUCUUGUAGUUGACTT
**siAtg16l1-2**	AGGGAAGAUCACUGCUCUGTT	CAGAGCAGUGAUCUUCCCUTT
**siAtg16l1-3**	UGGACACUCAUCCUGCUUCTT	GAAGCAGGAUGAGUGUCCATT
