# Supplementary material for: BRG1 attenuates colonic inflammation and tumorigenesis through autophagy-dependent oxidative stress sequestration
Source: Nat Commun. 2019 Oct 10;10:4614. doi: 10.1038/s41467-019-12573-z (PMC6787222; doi:10.1038/s41467-019-12573-z)
Supplement: Supplementary file 3 — Reporting Summary [file 41467_2019_12573_MOESM3_ESM.pdf]

nature research

Corresponding author(s): Wei-Qiang Gao

Last updated by author(s): Aug 19, 2019

Reporting Summary

Nature Research wishes to improve the reproducibility of the work that we publish. This form provides structure for consistency and transparency in reporting. For further information on Nature Research policies, see Authors & Referees and the Editorial Policy Checklist.

Statistics

For all statistical analyses, confirm that the following items are present in the figure legend, table legend, main text, or Methods section.

n/a	Confirmed	
☐	☒	The exact sample size (*n*) for each experimental group/condition, given as a discrete number and unit of measurement
☐	☒	A statement on whether measurements were taken from distinct samples or whether the same sample was measured repeatedly
☐	☒	The statistical test(s) used AND whether they are one- or two-sided<br>*Only common tests should be described solely by name; describe more complex techniques in the Methods section.*
☒	☐	A description of all covariates tested
☒	☐	A description of any assumptions or corrections, such as tests of normality and adjustment for multiple comparisons
☐	☒	A full description of the statistical parameters including central tendency (e.g. means) or other basic estimates (e.g. regression coefficient) AND variation (e.g. standard deviation) or associated estimates of uncertainty (e.g. confidence intervals)
☐	☒	For null hypothesis testing, the test statistic (e.g. *F*, *t*, *r*) with confidence intervals, effect sizes, degrees of freedom and *P* value noted<br>*Give P values as exact values whenever suitable.*
☒	☐	For Bayesian analysis, information on the choice of priors and Markov chain Monte Carlo settings
☒	☐	For hierarchical and complex designs, identification of the appropriate level for tests and full reporting of outcomes
☐	☒	Estimates of effect sizes (e.g. Cohen's *d*, Pearson's *r*), indicating how they were calculated

Our web collection on statistics for biologists contains articles on many of the points above.

Software and code

Policy information about availability of computer code

Data collection	The CHIP-seq data were generated by Illumina Hiseq 2000 platform ;<br>QRT-PCR was performed in a Bio-Rad C1000 thermal cycler machine;<br>The autophagosome were performed by transmission electron microscopy;<br>Micrographs were obtained with a FEI Tecnai G2 Spirit transmission electron microscope.
Data analysis	Photos were imaged and quantified by Image J software;<br>Analysis of RNA expression(qRT-PCR), weight-loss monitoring, IHC/IF quantification were performed by Graphpad PRISM7;<br>ChIP-Seq analysis was performed by Active Motif.

For manuscripts utilizing custom algorithms or software that are central to the research but not yet described in published literature, software must be made available to editors/reviewers. We strongly encourage code deposition in a community repository (e.g. GitHub). See the Nature Research guidelines for submitting code & software for further information.

Data

Policy information about availability of data

All manuscripts must include a data availability statement. This statement should provide the following information, where applicable:

- Accession codes, unique identifiers, or web links for publicly available datasets
- A list of figures that have associated raw data
- A description of any restrictions on data availability

The authors declare that all data supporting the findings in this study are available within the paper, Supplementary information and Source data. All data are available from the authors upon reasonable request. RNA-Seq and ChIP-Seq raw data have been deposited in the Gene Expression Omnibus (GEO) under accession number GEO: GSE112128 and GSE135041.

Field-specific reporting

Please select the one below that is the best fit for your research. If you are not sure, read the appropriate sections before making your selection.

☒ Life sciences ☐ Behavioural & social sciences ☐ Ecological, evolutionary & environmental sciences

For a reference copy of the document with all sections, see nature.com/documents/nr-reporting-summary-flat.pdf

Life sciences study design

All studies must disclose on these points even when the disclosure is negative.

Sample size	The sample sizes for the each experiments were described in the relevant figure legends of the paper
Data exclusions	No data were excluded from the analyses
Replication	All of attempts to replicate experiments were successful
Randomization	Mice used for the biological replication were from the same generation or as little mates, samples were allocated into experimental groups base on the genotype or properties.
Blinding	The investigators were blinded to the genotype of the sample or mouse model.

Reporting for specific materials, systems and methods

We require information from authors about some types of materials, experimental systems and methods used in many studies. Here, indicate whether each material, system or method listed is relevant to your study. If you are not sure if a list item applies to your research, read the appropriate section before selecting a response.

Materials & experimental systems

n/a	Involved in the study
☐	☒ Antibodies
☒	☐ Eukaryotic cell lines
☒	☐ Palaeontology
☐	☒ Animals and other organisms
☐	☒ Human research participants
☒	☐ Clinical data

Methods

n/a	Involved in the study
☐	☒ ChIP-seq
☒	☐ Flow cytometry
☒	☐ MRI-based neuroimaging

Antibodies

Antibodies used

BRG1(EPNCIR111A) Abcam Cat# ab110641(1:10000) ;
Caspase3(8G10) Cell Signaling Technology Cat# 9665(1:1000);
C-Caspase 3 Cell Signaling Technology Cat# 9661(1:1000);
PARP (46D11) Cell Signaling Technology Cat# 9532(1:1000);
Ki67(B56) BD Biosciences Cat# 550609(1:500);
EpCAM (G8.8) BD Biosciences Cat# 552370(1:500);
ZO1 Invitrogen Cat# 40-2200(1:500);
8-OHd G Abcam Cat# ab48508(1:500);
LC3A/B(D3U4C) Cell Signaling Technology Cat# 12741(1:1000);
ATG16L1(D6A5) Cell Signaling Technology Cat# 8089(1:1000);
PCNA Santa Cruz Biotechnology Cat# SC-7909(1:1000);
P-H3 Cell Signaling Technology Cat# 9701(1:1000);
E-Cadherin Cell Signaling Technology Cat# 3195T(1:1000);
DCLK1 Abcam Cat# ab31704(1:500);
Claudin1 Cell Signaling Technology Cat# 4933T (1:1000);
ChgA Abcam Cat# ab715(1:1000);
Hes1 Abcam Cat# ab71559(1:500) ;

Validation

Antibody validations were performed as described on the manufacturers' websites and were supported by multiple publications. Antibodies were further validated by using positive and negative controls in our studies.

Animals and other organisms

Policy information about studies involving animals; ARRIVE guidelines recommended for reporting animal research

Laboratory animals	C57BL/6 mice,both male and female, were used in this study, and described in the paper for detailes.
Wild animals	The study did not involved wild animals.
Field-collected samples	Study did not involve samples collected from the field.
Ethics oversight	All mice were maintained in a specific-pathogen-free (SPF) facility and all experimental procedures were approved by the institutional biomedical research ethics committee of the Shanghai Institutes for Biological Sciences or Institute of Zoology, Chinese Academy of Sciences.

Note that full information on the approval of the study protocol must also be provided in the manuscript.

Human research participants

Policy information about studies involving human research participants

Population characteristics	These patients were from a Chinese family with healthy parents and without a family history of specific diseases.
Recruitment	All patients information and samples were collected based on clinical requirements for diagnosis.
Ethics oversight	The study was approved by the ethics committee of the Shanghai Tenth People's Hospital (SHSY-IEC-pap-16-24). The use of pathological specimens as well as the review of all the pertinent patient records was approved by the institutional ethics review board.

Note that full information on the approval of the study protocol must also be provided in the manuscript.

ChIP-seq

Data deposition

☒ Confirm that both raw and final processed data have been deposited in a public database such as GEO.

☒ Confirm that you have deposited or provided access to graph files (e.g. BED files) for the called peaks.

Data access links<br>*May remain private before publication.*	https://www.ncbi.nlm.nih.gov/geo/query/acc.cgi?acc=GSE112128<br>https://www.ncbi.nlm.nih.gov/geo/query/acc.cgi?acc=GSE135041
Files in database submission	BRG1-CHIPseq<br>colon epithelial cells input DNA<br>H3K9ac-WT<br>H3K9ac-KO<br>H3K9ac-input
Genome browser session<br>(e.g. UCSC)	no longer applicable

Methodology

Replicates	The experiment did not have replicates.
Sequencing depth	H3K9ac-WT: reads:87784244;paired-end;length:300<br>H3K9ac-KO: reads:103916338;paired-end;length:300
Antibodies	BRG1(EPNCIR111A) Abcam Cat# ab110641; H3K9ac Abcam Cat#ab4441
Peak calling parameters	Peak detection was performed using the MACS (v2.1.1) peak finding algorithm with 0.01 set as the p-value cutoff.
Data quality	For all libraries, fastp/0.20 was used to assess quality of all libraries.
Software	Genomic data analysis: Cutadapt (v1.9.1);Trimmomatic (v0.35);Bowtie2 (v2.2.6);MACS (v2.1.1)
